# The Characteristics, Long-Term Outcomes, Risk Factors, and Antithrombotic Therapy in Chinese Patients With Atrial Fibrillation and Bioprosthetic Valves

**DOI:** 10.3389/fcvm.2021.665124

**Published:** 2021-06-10

**Authors:** Jiameng Ren, Yanmin Yang, Jun Zhu, Shuang Wu, Juan Wang, Han Zhang, Xinghui Shao

**Affiliations:** Emergency and Intensive Care Center, Fuwai Hospital, National Center for Cardiovascular Diseases, Chinese Academy of Medical Sciences and Peking Union Medical College, Beijing, China

**Keywords:** atrial fibrillation, bioprosthetic valves, outcomes, risk factors, antithrombotic status, dynamic changes of risk factor, surgical ablation of atrial fibrillation

## Abstract

**Introduction:** There were few data about the clinical profiles and long-term outcomes in Chinese patients with atrial fibrillation (AF) and bioprosthetic valves.

**Methods:** The retrospective study enrolled 903 patients with bioprosthetic valve replacement at our hospital and discharged with a diagnosis of AF from January 2010 to December 2018.

**Results:** The median age was 65.6 (61.9–69.1) years, and 548 (60.7%) patients were women. During a follow-up period of 3.84 (2.64–5.51) years, 68 (1.8 per 100 person-years) patients died, 81 (2.1 per 100 person-years) patients developed thromboembolism, and 23 (0.6 per 100 person-years) patients experienced major bleeding. The CHA_2_DS_2_-VASc score, as a categorical variable (low, moderate, or high risk), predicted the risk of thromboembolism with the C-statistic of 0.6 (95% CI: 0.511–0.689, *p* = 0.046). The incidence of the CHA_2_DS_2_-VASc score increment was 11.6 per 100 person-years, and the annual reclassification rate of stroke risk (from a low or moderate group to a higher group) was 12.7%. The current proportion of oral anticoagulants was 52.3, 59, and 63.2%, respectively, in the low, moderate, and high stroke risk groups. Age (OR: 1.04, 95% CI: 1.01–1.06, *p* = 0.01), left atrial size (OR: 1.05, 95% CI: 1.03–1.08, *p* < 0.001), and rheumatic heart disease (OR: 1.49, 95% CI: 1.05–2.10, *p* = 0.025) were positively associated with the use of oral anticoagulants. The history of chronic kidney disease (OR: 0.20, 95% CI: 0.05–0.76, *p* = 0.018), prior surgical ablation (OR: 0.33, 95% CI: 0.24–0.47, *p* < 0.001), and antiplatelet agent use (OR: 0.08, 95% CI: 0.05–0.13, *p* < 0.001) were inversely related to the use of oral anticoagulants. Higher admission estimated glomerular filtration rate (HR: 0.515, 95% CI: 0.311–0.853, *p* = 0.01), left ventricular ejection fraction (HR: 0.961, 95% CI: 0.931–0.992, *p* = 0.014), concomitant surgical ablation (HR: 0.348, 95% CI: 0.171–0.711, *p* = 0.004), and rheumatic heart disease history (HR: 0.515, 95% CI: 0.311–0.853, *p* = 0.01) were associated with a lower risk of death. Surgical ablation (HR: 0.263, 95% CI: 0.133–0.519, *p* < 0.001) and oral anticoagulants (HR: 0.587, 95% CI: 0.375–0.918, *p* = 0.019) were related to a lower risk of thromboembolism.

**Conclusion:** Chinese patients with AF and bioprosthetic valve(s) were relatively young and had a high prevalence of rheumatic heart disease with few comorbidities. The percentage of mitral bioprosthetic valve replacement was high. The proportion of concomitant surgical ablation or surgical left atrial appendage occlusion or exclusion was relatively low. The thromboembolic events were the major long-term adverse events. The anticoagulation therapy was underused in patients at moderate or high stroke risk. The CHA_2_DS_2_-VASc score was verified to be used for predicting stroke risk in this population. The stroke risk dynamically changed; it needed to be reestimated once the risk factor changed.

## Introduction

With an aging population, the incidence of valvular heart disease is gradually increasing, as the prevalence increases from 0.3% (0.2–0.3) of those 18–44 years old to 11.7% (11.0–12.5) of those aged 75 years and older (1). Besides, China has a large burden of rheumatic valvular heart disease (2). A hospital-based survey in China (3) reported that the prevalence of severe valve diseases was 1.8%. Due to the large population in China, the number of patients with valvular heart diseases who need interventions increases. Furthermore, the bioprosthetic valve (BPV) is preferred to be used in older patients for valve replacement.

Atrial fibrillation (AF) is the most common arrhythmia, a strong relation to aging. The prevalence of AF will increase with an expanding elderly population in China (4), and the estimated number of AF patients was projected to surpass 25 million by 2045 (5). Thus, the number of patients with BPV and AF is increasing. Previous studies focused on the management of AF and enrolled only a few patients with both AF and BPVs, and the sample size was small (6–16). The risk factors of thromboembolism and mortality in such population were also unknown. Although the CHA_2_DS_2_-VASc score was confirmed to be effective in these patients, the dynamic changes of the score were uncertain. Besides, little data could be found in China. Our study aimed to provide some data from the following aspects to improve management: (1) the clinical characteristics; (2) antithrombotic therapy status and factors associated with oral anticoagulation use; (3) long-term outcomes and factors related to mortality and thromboembolism; and (4) the utility of CHA_2_DS_2_-VASc score and its dynamic changes.

## Methods

### Patients

The retrospective study reviewed 1,289 patients who had left-sided BPV replacement (BVR) at Fuwai Hospital and discharged with AF diagnosis from January 2010 to December 2018. AF was confirmed by reviewing clinical records, electrocardiographic evidence, and electronic databases according to the International Classification of Disease, 9th or 10th Revision, ICD-9 427.3 or ICD-10 I48. Patients with an existing mechanical valve and missing data and who died within 3 months after discharge were excluded. The study was approved by the ethics committee of our hospital and obeyed the Declaration of Helsinki. All patients provided written informed consent.

The baseline demographic data, vital signs, comorbidities, surgery information, and echocardiography data at discharge were collected by reviewing their medical records. Comorbidities including hypertension, diabetes mellitus, heart failure, coronary artery disease, prior myocardial infarction, previous percutaneous coronary intervention, peripheral vascular disease, previous stroke/transient ischemic attack, rheumatic heart disease, dyslipidemia, chronic obstructive pulmonary disease/emphysema, prior major bleeding, chronic kidney disease, and surgical information were obtained based on the medical records. Height, weight, systolic blood pressure, diastolic blood pressure, heart rate, serum creatine, and echocardiography data were collected from the last medical records before discharge. The body mass index was calculated by dividing weight in kilograms by the square of height in meters. The left ventricular ejection fraction, the left ventricular end-diastolic diameter, and the left atrial size (the anteroposterior diameter of the left atrium) were recorded. The creatine at discharge was collected, and the estimated glomerular filtration rate (eGFR) was calculated according to the Xiangya equation (17). Surgery information consisted of the BPV position, tricuspid valve plasty, surgical ablation, surgical left atrial appendage occlusion or exclusion, and concomitant coronary artery bypass grafting (CABG). The CHA_2_DS_2_-VASc score (18) was calculated by giving two points to each patient of age ≥75 years and a history of thromboembolism and one point to each patient aged 65 to 74 years, history of hypertension, diabetes mellitus, congestive heart failure, vascular disease, and female sex. Patients were attributed into three stroke risk categories according to CHA_2_DS_2_-VASc score: low risk with CHA_2_DS_2_-VASc score of 0 in male or 1 in female; intermediate risk with CHA_2_DS_2_-VASc score 1 in male or 2 in female; and high risk with CHA_2_DS_2_-VASc score ≥ 2 in male or 3 in female.

### Outcomes

**Figure 1:**
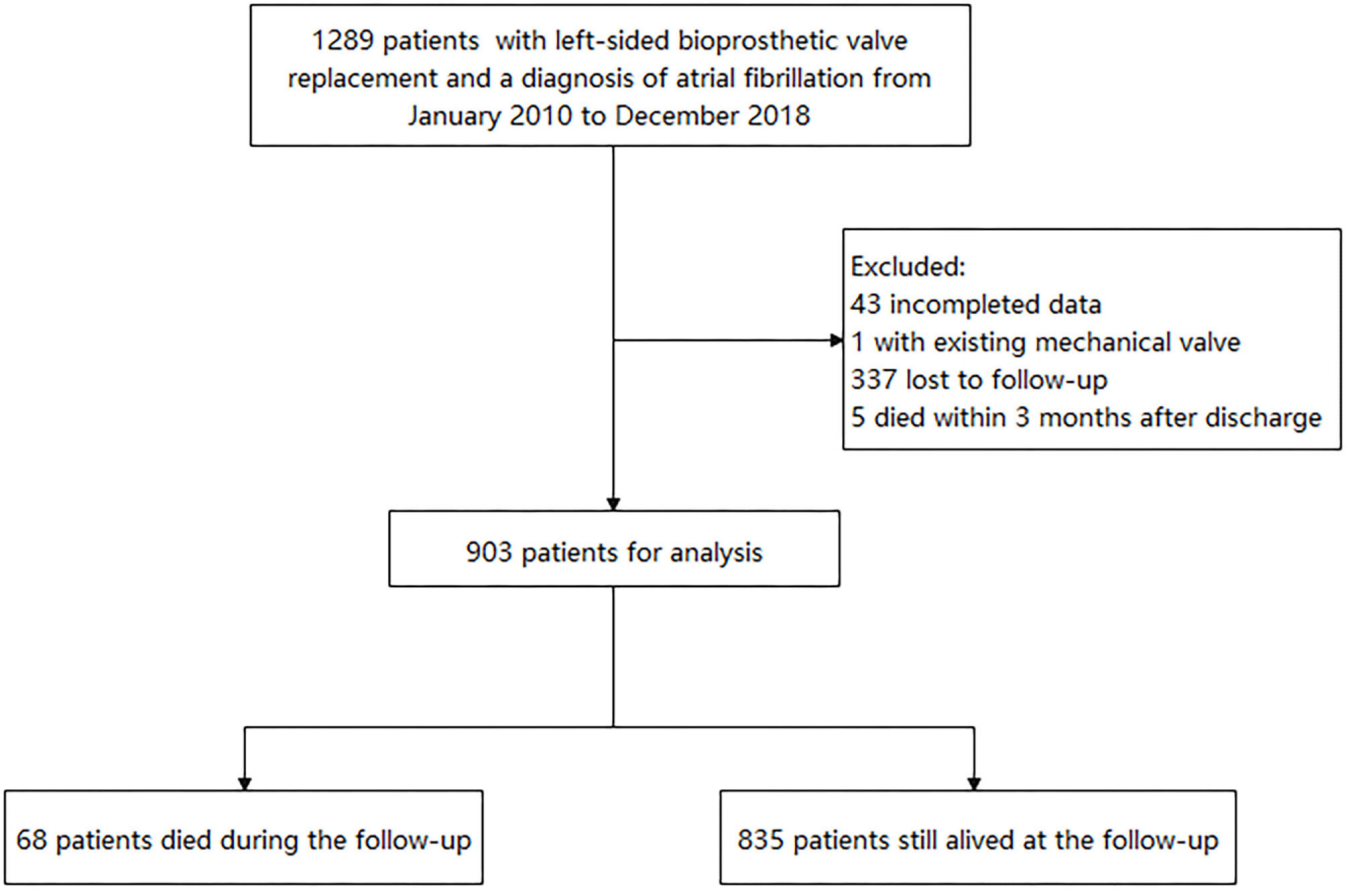
The flowchart.

Medical information after discharge was collected by reviewing the hospital electronic database or telephone interview until May 2020. Nevertheless, 337 patients had no outpatient or inpatient record at our hospital after the index hospitalization and could not be contacted. The flowchart is shown in Figure 1. The occurrence of stroke, noncentral nervous systemic embolism, pulmonary embolus, major bleeding, myocardial infarction, and death were collected. Since this was a retrospective study including data from a single center, there were possibilities that patients used outpatient services outside of the hospital. Thus, the adverse events, newly developed diseases, and antithrombotic agents were identified based on both the hospital electronic database records and patients' self-reports during telephone interviews. Trained physicians conducted the telephone interview. Thromboembolic events included ischemic stroke, noncentral nervous systemic embolism, or pulmonary embolus. Major bleeding was defined as the bleeding that led to hospitalization. The newly developed diseases (including hypertension, diabetes, heart failure, thromboembolic event, vascular diseases) were also recorded. The use of antithrombotic agents was defined as patients who still had oral anticoagulants or antiplatelet drugs (at least for 3 months) after the first 3 months of anticoagulated therapy for BPV replacement.

### Statistical Analysis

Categorical variables were expressed as frequencies and percentages, and continuous variables were expressed as means with standard deviations or medians with quartiles, depending on the distribution characteristics of variables. Between groups, comparisons were performed using Pearson's Chi-squared test or Fisher's exact test for categorical variables and the Student's *t*-test or the Mann–Whitney *U*-test for continuous variables. Kaplan–Meier survival curves were used to compare the event-free survival of the patients in the different groups. Cox regression analysis was performed to identify the factors related to thromboembolic events and all-cause death. Variables (not in CHA_2_DS_2_-VASc score) with a *P*-value < 0.05 in the univariate analysis, CHA_2_DS_2_-VASc score, and the use of oral anticoagulant (OAC) were entered into the multivariate Cox models for adjustment. Hazard ratio (HR) and 95% confidence interval (CI) were calculated. C-statistic was utilized to assess the performance of CHA_2_DS_2_-VASc score in patients not on anticoagulants. A logistic regression model was used to identify the factors related to the OAC use. The following variables were adjusted: age, female, body mass index, eGFR, hypertension, prior thromboembolism, diabetes, heart failure, coronary heart disease, peripheral vascular disease, rheumatic heart disease, major bleeding, chronic obstructive pulmonary disease/emphysema, chronic kidney disease, surgical ablation, surgical left atrial appendage occlusion or exclusion, concomitant CABG, and antiplatelet agent. Odds ratio (OR) and 95% CI were calculated.

The software package SPSS version 25.0 (IBM Corporation, New York, NY, USA) was used for statistical analysis. GraphPad Prism version 6.01 was utilized for figures. All statistical tests were two-tailed, and a *P*-value < 0.05 was considered significant.

## Results

### Baseline Characteristics

Finally, 903 patients were included in the present study for analysis. The baseline characteristics of patients are presented in Table 1. Five hundred forty-eight patients (60.7%) in the study were women. The median age was 65.6 (61.9–69.1) years, and the median body mass index was 23.8 (21.5–26.4) kg/m^2^. The eGFR was 61.2 ± 9.5 ml/min. The CHA_2_DS_2_-VASc score was 2.3 ± 1.4. Nearly half of patients (48%) were discharged with the CHA_2_DS_2_-VASc score ≥2 in men or ≥3 in women. The echocardiography presented a good cardiac function in the population with the median left atrial size of 44 (39–48) mm, median left ventricular ejection fraction of 60 (56–63) %, and median left ventricular end-diastolic diameter of 46 (42–49) mm. The prevalence of rheumatic heart disease was high (63%). The other frequently reported comorbidities were hypertension (29.7%), coronary artery disease (22.1%), dyslipidemia (16.3%), previous stroke or transient ischemic attack (14.2%), and diabetes mellitus (12.5%). Regarding the surgical information, 642 patients (71.1%) had mitral BVR, 112 patients (12.4%) had aortic BVR, 144 patients (15.9%) had both mitral and aortic BVR, three patients (0.3%) had both mitral and tricuspid BVR, and two patients had mitral, aortic, and tricuspid BVR. Regarding the concomitant surgery, surgical ablation was performed in 287 (31.8%) patients, surgical left atrial appendage occlusion or exclusion was done for 131 (14.5%) patients, and CABG was done in 175 (19.4%) patients. The statistical difference between patients included and those not included was reached in age, body mass index, CHA_2_DS_2_-VASc score, previous stroke/transient ischemic attack, BPV position, and surgical left atrial appendage occlusion or exclusion (Supplementary Table 1).

**Table 1:** Baseline characteristics of patients.

**Variables**	**Total**
	***n* = 903**
**Demographics**	
Male, *n* (%)	355 (39.3%)
Age, years	65.3 ± 6.6
BMI, kg/m^2^	24 ± 3.5
SBP, mmHg	115.4 ± 11.7
DBP, mmHg	69.1 ± 8.8
HR, bpm	83.8 ± 15.4
eGFR, ml/min	61.2 ± 9.5
CHA_2_DS_2_-VASc score	2.3 ± 1.4
Left atrial size, mm	44.2 ± 7.8
Left ventricular ejection fraction, %	59.2 ± 6.7
Left ventricular end diastolic diameter, mm	46 ± 5.9
**Medical history**, ***n*** **(%)**	
Hypertension	268 (29.7%)
Heart failure	68 (7.5%)
Coronary artery disease	200 (22.1%)
Previous myocardial infarction	15 (1.7%)
Previous percutaneous coronary intervention	19 (2.1%)
Previous stroke or TIA	130 (14.4%)
Diabetes mellitus	113 (12.5%)
Peripheral vascular disease	77 (8.5%)
Rheumatic heart disease	569 (63%)
Chronic obstructive pulmonary disease/emphysema	16 (1.8%)
Chronic kidney disease	14 (1.6%)
Dyslipidemia	147 (16.3%)
Major bleeding	10 (1.1%)
**Surgical information**, ***n*** **(%)**	
Bioprosthetic valve position	
Aortic alone	112 (12.4%)
Mitral and aortic	144 (15.9%)
Mitral alone	642 (71.1%)
Mitral and tricuspid	3 (0.3%)
Mitral, aortic, and tricuspid	2 (0.2%)
Tricuspid valve plasty	576 (63.8%)
Surgical ablation	287 (31.8%)
Surgical left atrial appendage occlusion or exclusion	131 (14.5%)
Concomitant coronary artery bypass grafting	175 (19.4%)

*BMI, body mass index; SBP, systolic blood pressure; DBP, diastolic blood pressure; HR, heart rate; eGFR, estimated glomerular filtration rate; TIA, transient ischemic attack*.

### Outcomes

The median follow-up period was 3.84 (2.64–5.51) years. The incidence of adverse events in patients with or without OAC is shown in Table 2. Sixty-eight (1.8 per 100 person-years) patients died. Among them, 28 (41.2%) patients died due to cardiovascular diseases, 16 (23.5%) was due to thromboembolism, and 6 (8%) patients died of major bleeding. The event-free survival of the patients in the different groups is illustrated in Supplementary Figure 1.

**Table 2:** The outcomes of patients with OAC (*n* = 538) or without OAC (*n* = 365) during the follow-up.

**Outcomes**	**Total**	**Stroke risk category at baseline**			**Surgical ablation**		**Rheumatic heart disease**		**Surgical left atrial appendage occlusion or exclusion**	
	**No. event (yearly rate, %)**	**Low**	**Moderate**	**High**	**Yes**	**No**	**Yes**	**No**	**Yes**	**No**
Thromboembolism	81 (2.1)	10 (1.1)	28 (2.3)	43 (2.4)	10 (0.8)	71 (2.7)	54 (2.2)	27 (1.9)	5 (1.2)	76 (2.2)
No OAC	37 (2.5)	4 (1)	12 (2.7)	21 (3.4)	5 (0.7)	32 (4.3)	19 (2.2)	18 (2.8)	2 (1)	35 (2.7)
OAC	44 (1.9)	6 (1.3)	16 (2.3)	22 (2)	5 (1)	39 (2.2)	35 (2.3)	9 (1.2)	3 (1.4)	41 (2)
All-cause death	68 (1.8)	8 (0.9)	20 (1.7)	40 (2.2)	9 (0.7)	59 (2.3)	31 (1.2)	37 (2.7)	5 (1.2)	63 (1.8)
No OAC	20 (1.4)	1 (0.2)	4 (0.9)	15 (2.4)	4 (0.5)	16 (2.1)	8 (0.9)	12 (1.9)	0 (0)	20 (1.6)
OAC	48 (2.1)	7 (1.5)	16 (2.3)	25 (2.3)	5 (1)	43 (2.5)	23 (1.5)	25 (3.5)	5 (2.4)	43 (2.1)
Major bleeding	23 (0.6)	2 (0.2)	6 (0.5)	15 (0.8)	4 (0.3)	13 (0.5)	9 (0.4)	14 (1)	0 (0)	17 (0.5)
No OAC	8 (0.5)	1 (0.2)	2 (0.4)	5 (0.8)	2 (0.3)	6 (0.8)	4 (0.5)	4 (0.6)	0 (0)	8 (0.6)
OAC	15 (0.7)	1 (0.2)	4 (0.6)	10 (0.9)	2 (0.4)	13 (0.7)	5 (0.3)	10 (1.4)	0 (0)	15 (0.7)
Myocardial infarction	6 (0.2)	2 (0.2)	3 (0.3)	1 (0.1)	0 (0)	6 (0.2)	3 (0.1)	3 (0.2)	0 (0)	6 (0.2)
No OAC	2 (0.1)	0 (0)	1 (0.2)	1 (0.2)	0 (0)	2 (0.3)	0 (0)	2 (0.3)	0 (0)	2 (0.2)
OAC	4 (0.2)	2 (0.4)	2 (0.3)	0 (0)	0 (0)	4 (0.2)	3 (0.2)	1 (0.1)	0 (0)	4 (0.2)

*OAC, oral anticoagulant*.

The factors associated with all-cause mortality and thromboembolic events are presented in Table 3 and Table 4. Regarding the all-cause mortality, patients with low or moderate stroke risk at baseline had a lower incidence during the follow-up than the high-risk groups, whereas the difference was not seen after adjustment (*p* = 0.782). Patients with higher admission eGFR, higher left ventricular ejection fraction, concomitant surgical ablation, or rheumatic heart disease history had a significantly lower risk of death. Moreover, the results persisted in the multivariable model. As for thromboembolic events, only surgical ablation and the use of OAC significantly associated with a lower risk of thromboembolism.

**Table 3:** The factors associated with all-cause death in patients with atrial fibrillation and bioprosthetic valve (*n* = 903).

**Variables**	**Univariable Cox regression analysis**		Multivariable Cox regression model^*^	
	***P*-value**	**HR (95% CI)**	***P*-value**	**HR (95% CI)**
Age	0.002	1.07 (1.03–1.11)		
Female	0.002	0.47 (0.29–0.76)		
CHA_2_DS_2_-VASc score	0.041		0.782	
Low		Reference		Reference
Intermediate	0.111	1.95 (0.86–4.43)	0.515	1.32 (0.57–3.05)
High	0.013	2.61 (1.22–5.59)	0.756	1.15 (0.49–2.71)
Hypertension	0.538	1.18 (0.70–1.97)		
Previous stroke or TIA	0.595	1.19 (0.63–2.21)		
Diabetes	0.575	1.21 (0.62–2.37)		
Heart failure	0.053	2.08 (0.99–4.37)		
Coronary heart disease	0.037	1.73 (1.03–2.89)		
Peripheral vascular disease	0.045	2.14 (1.02–4.50)		
Surgical LAAO	0.671	0.82 (0.33–2.06)		
OAC use during follow-up	0.091	1.57 (0.93–2.65)		
eGFR	0.045	0.98 (0.95–1.00)	0.005	0.97 (0.94–0.99)
Left ventricular ejection fraction, %	0.001	0.95 (0.92–0.98)	0.016	0.96 (0.93–0.99)
Rheumatic heart disease	0.002	0.47 (0.29–0.76)	0.01	0.52 (0.31–0.85)
Surgical ablation	0.001	0.31 (0.15–0.63)	0.004	0.35 (0.17–0.71)
CABG	0.005	2.11 (1.26–3.53)	0.197	1.87 (0.72–4.83)

*HR, hazard ratio; CI, confidence interval; TIA, transient ischemic attack; LAAO, left atrial appendage occlusion or exclusion; OAC, oral anticoagulant; eGFR, estimated glomerular filtration rate; CABG, concomitant coronary artery bypass grafting*.

* *Adjusted for CHA_2_DS_2_-VASc score (three categories: low, moderate, high), eGFR, left ventricular ejection fraction, rheumatic heart disease, surgical ablation, CABG*.

**Table 4:** The factors associated with thromboembolism in patients with atrial fibrillation and bioprosthetic valve (*n* = 903).

**Variables**	**Univariable Cox regression analysis**		Multivariable Cox regression model^*^	
	***P*-value**	**HR (95% CI)**	***P*-value**	**HR (95% CI)**
Age	0.023	1.04 (1.01–1.08)		
Female	0.563	0.88 (0.56–1.37)		
eGFR	0.657	1.00 (0.97–1.018)		
Left ventricular ejection fraction, %	0.449	1.01 (0.98–1.05)		
Heart failure	0.946	0.97 (0.39–2.40)		
Coronary heart disease	0.335	1.28 (0.77–2.13)		
Rheumatic heart disease	0.501	1.17 (0.74–1.86)		
Peripheral vascular disease	0.179	1.65 (0.79–3.44)		
Hypertension	0.204	1.35 (0.85–2.13)		
Previous stroke or TIA	0.54	0.81 (0.42–1.58)		
Major bleeding	0.533	0.05 (0.00–651.34)		
Diabetes	0.489	1.24 (0.67–2.29)		
Chronic kidney disease	0.882	0.86 (0.12–6.20)		
Surgical LAAO	0.234	0.58 (0.23–1.43)		
Tricuspid valve plasty	0.173	1.39 (0.86–2.25)		
CHA_2_DS_2_-VASc score	0.062		0.272	
Low		Reference		Reference
Intermediate	0.036	2.16 (1.05–4.45)	0.109	1.81 (0.88–3.76)
High	0.021	2.25 (1.13–4.48)	0.181	1.65 (0.79–3.42)
Surgical ablation	<0.001	0.28 (0.14–0.54)	<0.001	0.26 (0.13–0.52)
CABG	0.042	1.68 (1.02–2.76)	0.3	1.33 (0.77–2.30)
OAC use during follow-up	0.243	0.77 (0.50–1.19)	0.019	0.59 (0.38–0.92)

*HR, hazard ratio; CI, confidence interval; TIA, transient ischemic attack; eGFR, estimated glomerular filtration rate; COPD, chronic obstructive pulmonary disease; LAAO, left atrial appendage occlusion or exclusion; CABG, concomitant coronary artery bypass grafting; OAC, oral anticoagulant*.

* *Adjusted for CHA_2_DS_2_-VASc score (three categories: low, moderate, high), surgical ablation, CABG, OAC use during the follow-up*.

### Stroke Risk Prediction and the Change of Stroke Risk Profile

There were 365 patients not on anticoagulants during the follow-up. The C-statistic of CHA_2_DS_2_-VASc score in predicting thromboembolism in patients without OAC was 0.585 as a continuous variable (95% CI: 0.493–0.677, *p* = 0.089) and 0.6 as categorical variable (95% CI: 0.511–0.689, *p* = 0.046, categories: low, moderate, and high risk). The annual rate of thromboembolism in the low-, moderate-, and high-risk groups was 0.97, 2.66, and 3.41%, respectively.

The changes in stroke risk profile were analyzed in patients alive at the follow-up visit (*n* = 835). During the follow-up, the number and annual incidence of hypertension, diabetes, heart failure, thromboembolic event, vascular disease were 52 (1.4%), 19 (0.5%), 49 (1.4%), 59 (1.6%), and 2 (0.1%), respectively. The mean age increased from 65.1 ± 6.6 to 69.0 ± 6.8 years old. The mean CHA_2_DS_2_-VASc score increased from 2.2 ± 1.4 at baseline to 2.9 ± 1.5 at the end of follow-up period. There were 418 (50.1%) patients had an increase of CHA_2_DS_2_-VASc score, and the incidence of CHA_2_DS_2_-VASc score increment was 11.6 per 100 person-years. Moreover, 330 patients had one-point increment due to age. The change in the stroke risk category is shown in Supplementary Figure 2. A total of 249 patients who were at low or intermediate risk at baseline were reclassified into a higher-risk category, and the reclassification rate was 12.7 per 100 person-years.

### The Antithrombotic Status and Factors Related to OAC Use

**Figure 2:**
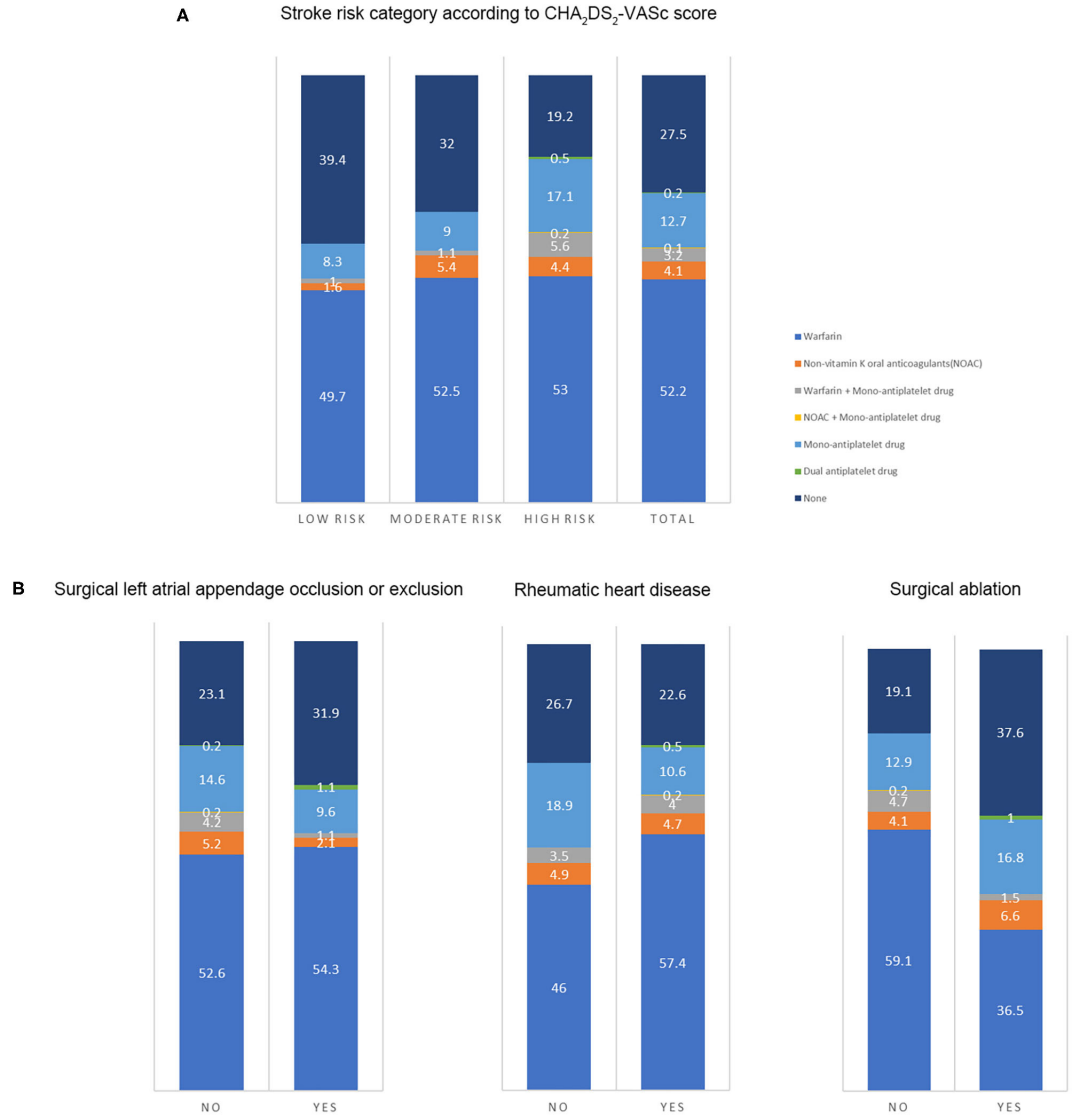
The antithrombotic therapy in patients. The number on the figure was the percentage of therapies in each group. **(A)** Antithrombotic therapy in the total population and patients at different stroke risk (*n* = 903) (Low risk: CHA_2_DS_2_-VASc score of 0 in male or 1 in female; intermediate risk: CHA_2_DS_2_-VASc score of 1 in male or 2 in female; high risk: CHA_2_DS_2_-VASc score ≥ 2 in male or 3 in female). **(B)** The antithrombotic agents in patients at moderate or high stroke risk in different groups (*n* = 710) (With or without prior surgical left atrial appendage occlusion or exclusion; with or without a history of rheumatic heart disease; with or without a history of surgical ablation).

The antithrombotic therapy status in patients still alive is presented in Figure 2A. The percentage of OAC use was high in patients with low stroke risk (52.3%), and the OAC was underused in patients at moderate (59%) or high risk (63.2%). The percentage of non-vitamin K antagonist OACs was low in such population (4.2%). The antithrombotic agents in patients at moderate or high stroke risk in different groups (*n* = 710) are shown in Figure 2B. The proportion of OAC use in patients with or without surgical left atrial appendage occlusion or exclusion was similar (57.4 vs. 62.2%, *p* = 0.38). The percentage in patients with surgical ablation significantly differed from that in those without the surgery (44.7 vs. 68%, *p* < 0.001). Patients with a history of rheumatic heart disease took OAC more frequently than those without (66.4 vs. 54.4%, *p* = 0.001). Considering the factors related to OAC use, age, left atrial size, and rheumatic heart disease were positively associated with OAC use. Nevertheless, the history of chronic kidney disease, prior surgical ablation, and antiplatelet agent use were inversely related to the OAC use (Table 5).

**Table 5:** The factors related to the use of OAC.

**Variable**	***P*-value**	OR (95% CI)^*^
Age	0.01	1.04 (1.01–1.06)
Female	0.814	1.04 (0.74–1.48)
Body mass index	0.346	1.02 (0.98–1.07)
eGFR	0.096	0.99 (0.97–1.00)
Left atrial size	<0.001	1.05 (1.03–1.08)
LVEF	0.215	1.02 (0.99–1.05)
Hypertension	0.56	1.12 (0.77–1.61)
Heart failure	0.185	1.63 (0.79–3.33)
Coronary heart disease	0.052	2.01 (0.99–4.06)
Previous stroke or TIA	0.163	1.39 (0.88–2.21)
Rheumatic heart disease	0.025	1.49 (1.05–2.10)
Diabetes	0.988	1.00 (0.62–1.61)
Peripheral vascular disease	0.947	0.98 (0.55–1.75)
Major bleeding	0.475	1.73 (0.39–7.74)
Chronic obstructive pulmonary disease/emphysema	0.895	0.92 (0.27–3.13)
Chronic kidney disease	0.018	0.20 (0.05–0.76)
Concomitant CABG	0.821	0.92 (0.44–1.92)
Surgical ablation	<0.001	0.33 (0.24–0.47)
Surgical left atrial appendage occlusion or exclusion	0.361	0.82 (0.53–1.26)
Antiplatelet agent	<0.001	0.08 (0.05–0.13)

*OR, odds ratio; CI, confidence interval; eGFR, estimated glomerular filtration rate; LVEF, left ventricular ejection fraction; TIA, transient ischemic attack; CABG, coronary artery bypass graft; left atrial size: the anteroposterior diameter of the left atrium*.

* *Calculated by multivariable logistic regression model. The following variables were adjusted: age, female, body mass index, eGFR, left atrial size, LVEF, hypertension, heart failure, coronary heart disease, prior thromboembolism, rheumatic heart disease, diabetes, peripheral vascular disease, major bleeding, lung disease, chronic kidney disease, concomitant CABG, surgical ablation, surgical left atrial appendage occlusion or exclusion, antiplatelet agent*.

## Discussion

The present study presented a profile of Chinese patients with AF and BPVs, including the clinical characteristics, long-term outcomes, risk factors, and current antithrombotic status. Chinese patients with AF and BPV(s) were relatively young and had a high prevalence of rheumatic heart disease with few comorbidities. The percentage of mitral BPV replacement was high. The proportion of concomitant surgical ablation or surgical left atrial appendage occlusion or exclusion was relatively low. The thromboembolic events were the major long-term adverse events and needed more attention. The anticoagulation therapy was underused in patients at moderate or high stroke risk. The CHA_2_DS_2_-VASc score was verified to be used for predicting stroke risk in this population. Also, the thromboembolic risk dynamically changed; it needed to be re-estimated once the risk factor changed. We also found that surgical ablation was associated with a lower incidence of all-cause mortality and thromboembolism. The study filled the lack of data on Chinese patients with BPV and AF, especially those with a history of rheumatic heart disease, and further provided some information to help improve the management in such patients.

The clinical characteristics in our study were different from those reported in previous studies (6, 7, 10, 11). Patients were younger in our study than those in previous studies. There were more women and a high prevalence of rheumatic heart disease. Besides, the comorbidities were also not as much as other literature reported. Thus, the CHA_2_DS_2_-VASc score was relatively lower compared to existing data. The studies also showed a very high proportion (nearly 90%) of mitral BPV. The related reasons might be as follows: first, the enrollment in the present study was based on the patients who underwent BVR at our hospital and discharged with AF diagnosis. The patients who developed AF after the BPV were not included, which was the enrollment criteria for the BPV-AF study (7), and these patients were generally older and had more comorbidities than patients in our study. Second, according to the Cardiovascular Surgery Outcomes 2019 published by Fuwai Hospital (19), which is the largest medical center for cardiovascular diseases in China, the proportion of the application of BPVs was only 20%, significantly lower than that in other countries. Third, considering the need for anticoagulation in AF patients, some patients preferred to choose the mechanical valve when undergoing valve replacement. Furthermore, the most common cause of severe mitral stenosis remained rheumatic fever in China (3), and such patients needed valve replacement at a relatively young age. In that condition, these patients did not suffer from many comorbidities as our studies depicted. Even so, our study could provide information in patients with AF and BPV, especially for AF patients who underwent BPV replacement due to rheumatic valvular diseases.

The present study validated the prediction utility of CHA_2_DS_2_-VASc score in non-anticoagulated Chinese AF patients with BPVs. The result was in line with that reported by Philippart et al. in 2016 (20), in which C-statistic was 0.554 (0.475–0.632) as a continuous variable and 0.598 (0.519–0.674) as a categorical variable. The CHA_2_DS_2_-VASc score only has a modest ability in predicting stroke risk. According to our results, the score had a good performance in identifying the low-risk group. Nevertheless, the incidence of stroke in moderate- or high-risk groups was comparable and high, which the anticoagulants needed to be considered. The dynamic changes of CHA_2_DS_2_-VASc score were also assessed. The annual increment (11.6% per 100 person-years) was similar to that previously reported (12.1% per 100 person-years) (21). In our study, 12.7% of patients in a low or moderate stroke risk group could progress to higher risk groups every year. Moreover, 80% of new comorbidities were evident at 4.2 months after AF was diagnosed (21), and annual ischemic stroke rates were significantly higher in the reclassified “intermediate” or “high-risk” groups than the unchanged groups (22). Thus, it is essential to re-estimate the risk in those patients frequently.

The anticoagulated status and strategy in AF patients with BPVs for stroke prevention need to be improved. Previous studies mainly focused on the early antithrombotic therapy in patients who underwent BPV replacemen (23, 24), but few papers paid attention to the long-term antithrombotic status and strategy in patients with BPV and AF. The proportion of oral anticoagulation in such Chinese patients was low. Furthermore, 5.6% of patients had both OAC and mono-antiplatelet drug, which increased bleeding risk (23). Patients with a CHA_2_DS_2_-VASc score of 0 or 1 had a relatively high anticoagulated proportion. The history of rheumatic heart disease in our study might contribute to the phenomena in the low-risk group. On the one hand, the unsatisfactory anticoagulated status was a Chinese national condition (25). On the other hand, few studies investigated the anticoagulation strategy in patients with BPV and AF, especially those who previously suffered from rheumatic heart diseases. Recently, some studies investigated the effect of non-vitamin K antagonist OACs in such patients and found that non-vitamin K antagonist OACs might also be used in such population with similar effectiveness and less bleeding risk compared with warfarin (26). The annual thromboembolic rate was high in the anticoagulated population, which indicated that the quality of anticoagulation therapy should also be improved. The median time in the therapeutic range was only 51.7% in Chinese patients according to the CAFR study (27, 28), which enrolled consecutive AF patients from 32 tertiary and non-tertiary hospitals in Beijing, China. The time in the therapeutic range might be overestimated since Beijing has the best medical resources in China. More attention should be paid to the adherence to the guideline and quality of anticoagulation.

The risk factors of all-cause mortality and thromboembolic events were explored. As for all-cause mortality, rheumatic heart disease and surgical ablation were proved to be protective factors in addition to the most studied risk factors, including age, female, coronary heart disease, peripheral vascular disease, left ventricular ejection fraction, and eGFR. Regarding the surgical ablation, several studies showed that surgical ablation significantly reduced mortality during short- or long-term follow-up (29–31), and similar results were seen in our study. The low incidence of death in patients with rheumatic heart disease might relate to the better baseline characteristics in these patients, that they were younger and have fewer comorbidities. As for the thromboembolic risk, our results showed that surgical ablation was significantly associated with lower incidence of thromboembolic events as well as OAC use. Nevertheless, the effect of surgical ablation on thromboembolism was not consistent. A meta-analysis published in 2018, including 23 randomized controlled trials with small sample sizes, found no impact of surgical ablation on mortality and stroke (32). The follow-up periods in most of the studies were 1 year and no more than 2 years. Recently, a large study (33) based on the medical database published in 2019 showed that concomitant ablation in CABG patients with preoperative AF was associated with lower stroke or systemic embolization [HR 0.73 (0.61–0.87), *p* = 0.0006]. In the same year, according to the results from the PRAGUE-12 Study, which was a prospective study, the randomized clinical trial enrolled 207 patients and had a 5-year follow-up, demonstrating that concomitant surgical ablation of AF was associated with a decreased risk of stroke [subhazard ratio 0.32 (0.12–0.84), *p* = 0.02] (34). Further, Kim et al. (35) reported that surgical AF ablation during rheumatic mitral valve surgery was associated with a lower risk of long-term mortality and thromboembolic events. Similarly, most of the patients in our study had a history of rheumatic heart disease. Also, recent evidence found that rhythm control could improve symptom and reduce adverse events (36, 37). Above all, our study verified the results in a Chinese population with a long-term follow-up and supported the surgical ablation procedure concomitant to other cardiac surgeries. As for the surgical left atrial appendage occlusion/exclusion, the results from multiple small sample size studies (38–40) indicated a decreased risk of stroke in patients with the surgery. Although patients in the surgical left atrial appendage occlusion/exclusion group had a lower incidence of thromboembolism, no statistical significance was seen in our study. On the one hand, patients were at low or moderate risk of stroke with a mean CHA_2_DS_2_-VASc score of 2.3. The impact of the surgery might be attenuated due to the low incidence in such a population. On the other hand, only 132 patients in our study underwent the surgical left atrial appendage occlusion/exclusion. The small sample size might limit to get the statistical significance. Besides, anticoagulation therapy could mask its effect. Further investigations are needed.

### Limitations

There were several limitations in our study. First, this was a retrospective study with its inherent defects; our results should be considered with caution and as hypothesis-generating. Second, the study only included data from one single center. There were possibilities that patients used outpatient services outside of the hospital. Considering this, the adverse events, drugs, and newly developed diseases were identified based on both the hospital electronic database records and patients' self-reports during telephone interviews. Besides, our hospital is the national center for cardiovascular diseases and has the largest cardiac procedure volume in China. This study, which focused on patients with AF and BPV, had a relatively large sample size so far to the best of our knowledge. Third, patients who developed AF after discharging from index hospital were not included. The previous study enrolled few patients with a history of rheumatic valvular diseases, while two-thirds of the patients in our study had rheumatic valvular diseases. In that case, we filled the gaps in such a specific population. Fourth, data on specific surgical procedures and the recurrences of AF were not collected. In addition, we could not assess the application of non-vitamin K OACs, and further prospective studies are needed.

## Conclusion

The hospital-based study presented a profile of Chinese patients with AF and BPVs, including the clinical characteristics, outcomes, risk factors, the change of stroke risk, and the current antithrombotic status. The study provided data for physicians to improve the management in such patients. Further prospective studies are needed to validate the results in our study.

## Data Availability Statement

The raw data supporting the conclusions of this article will be made available by the authors, without undue reservation.

## Ethics Statement

The studies involving human participants were reviewed and approved by the ethics committee of Fuwai hospital. The patients/participants provided their written informed consent to participate in this study.

## Author Contributions

YY and JR conceived the present study and participated in the design. JR conducted the data analysis and drafted the manuscript. JR, SW, HZ, XS, and JW collected and assembled all the data. YY and JZ commented on the manuscript drafts. All authors contributed to the article and approved the submitted version.

## Conflict of Interest

The authors declare that the research was conducted in the absence of any commercial or financial relationships that could be construed as a potential conflict of interest.

## Supplementary Material

Supplementary Figure 1 The event-free survival of the patients in the different groups (*n* = 903). **(A)** All-cause death; **(B)** Thromboembolism.

Supplementary Figure 2 The change of the stroke risk category (*n* = 835).

## Abbreviations

AF: Atrial fibrillation
BVR: Bioprosthetic valve replacement
BPV: Bioprosthetic valve
eGFR: estimated glomerular filtration rate
CABG: coronary artery bypass grafting
OAC: oral anticoagulant
HR: hazard ratio
CI: confidence interval.
