# Supplementary material for: The Characteristics, Long-Term Outcomes, Risk Factors, and Antithrombotic Therapy in Chinese Patients With Atrial Fibrillation and Bioprosthetic Valves
Source: Front Cardiovasc Med. 2021 Jun 10;8:665124. doi: 10.3389/fcvm.2021.665124 (PMC8222517; doi:10.3389/fcvm.2021.665124)
Supplement: Supplementary file 1 [file Table_1.DOCX]

Table S1. The baseline characteristics between patients included and these not included

| Variables | Total(n=1289) | Included(n=903) | Excluded(n=386) | P value |
| --- | --- | --- | --- | --- |
| Demographics |  |  |  |  |
| Male,n(%) | 524(40.7%) | 355(39.3%) | 169(43.8%) | 0.135 |
| Age,years | 65±6.8 | 65.3±6.6 | 64.3±7.1 | 0.015 |
| BMI,kg/m2 | 23.8±3.5 | 24±3.5 | 23.2±3.3 | <0.001 |
| SBP,mmHg | 115.2±11.5 | 115.4±11.7 | 114.7±11.1 | 0.363 |
| DBP,mmHg | 68.9±8.7 | 69.1±8.8 | 68.5±8.4 | 0.271 |
| HR,bpm | 83.6±15.2 | 83.8±15.4 | 83±14.6 | 0.379 |
| eGFR,ml/min | 61.4±9.4 | 61.2±9.5 | 62±9.2 | 0.168 |
| CHA_2_DS_2_ -VAS score | 2.2±1.3 | 2.3±1.4 | 2±1.3 | 0.001 |
| Left atrial size,mm | 44.2±8 | 44.2±7.8 | 44.3±8.5 | 0.689 |
| LVEF,% | 59±6.9 | 59.2±6.7 | 58.6±7.3 | 0.134 |
| LVEDD,mm | 46±6.1 | 46±5.9 | 46.2±6.6 | 0.873 |
| Medical history,n(%) |  |  |  |  |
| Hypertension | 370(28.7%) | 268(29.7%) | 102(26.4%) | 0.237 |
| Heart failure | 90(7%) | 68(7.5%) | 22(5.7%) | 0.237 |
| Coronary artery disease | 278(21.6%) | 200(22.1%) | 78(20.2%) | 0.438 |
| Previous myocardial infarction | 17(1.3%) | 15(1.7%) | 2(0.5%) | 0.09 |
| Previous PCI | 22(1.7%) | 19(2.1%) | 3(0.8%) | 0.092 |
| Previous stroke or TIA | 169(13.1%) | 130(14.4%) | 39(10.1%) | 0.036 |
| Diabetes mellitus | 161(12.5%) | 113(12.5%) | 48(12.4%) | 0.969 |
| Peripheral vascular disease | 103(8%) | 77(8.5%) | 26(6.7%) | 0.277 |
| Rheumatic heart disease | 804(62.4%) | 569(63%) | 235(60.9%) | 0.469 |
| Lung diseases | 22(1.7%) | 16(1.8%) | 6(1.6%) | 0.782 |
| Chronic kidney disease | 21(1.6%) | 14(1.6%) | 7(1.8%) | 0.733 |
| Dyslipidemia | 196(15.2%) | 147(16.3%) | 49(12.7%) | 0.101 |
| Major bleeding | 17(1.3%) | 10(1.1%) | 7(1.8%) | 0.309 |
| Surgery information,n(%) |  |  |  |  |
| Bioprosthetic valve position | |  |  | 0.02 |
| Aortic alone | 153(11.9%) | 112(12.4%) | 41(10.6%) |  |
| Mitral and aortic | 230(17.8%) | 144(15.9%) | 86(22.3%) |  |
| Mitral alone | 896(69.5%) | 642(71.1%) | 254(65.8%) |  |
| Mitral and tricuspid and/or aortic | 10(0.8%) | 5(0.6%) | 5(1.3%) |  |
| Tricuspid valve plasty | 828(64.2%) | 576(63.8%) | 252(65.3%) | 0.607 |
| Surgical radiofrequency ablation | 389(30.2%) | 287(31.8%) | 102(26.4%) | 0.055 |
| Surgical left atrial appendage occlusion or exclusion | 164(12.7%) | 131(14.5%) | 33(8.5%) | 0.003 |
| Concomitant CABG | 249(19.3%) | 175(19.4%) | 74(19.2%) | 0.931 |

BMI: Body mass index; SBP: Systolic blood pressure; DBP: Diastolic blood pressure; HR: Heart rate; eGFR: estimated glomerular filtration rate; TIA: Transient ischemic attack.;LVEF: Left ventricular ejection fraction; LVEDD: Left ventricular end diastolic diameter; CABG:Coronary artery bypass grafting.
