# Supplementary figures and images for: The Characteristics, Long-Term Outcomes, Risk Factors, and Antithrombotic Therapy in Chinese Patients With Atrial Fibrillation and Bioprosthetic Valves
Source: Front Cardiovasc Med. 2021 Jun 10;8:665124. doi: 10.3389/fcvm.2021.665124 (PMC8222517; doi:10.3389/fcvm.2021.665124)

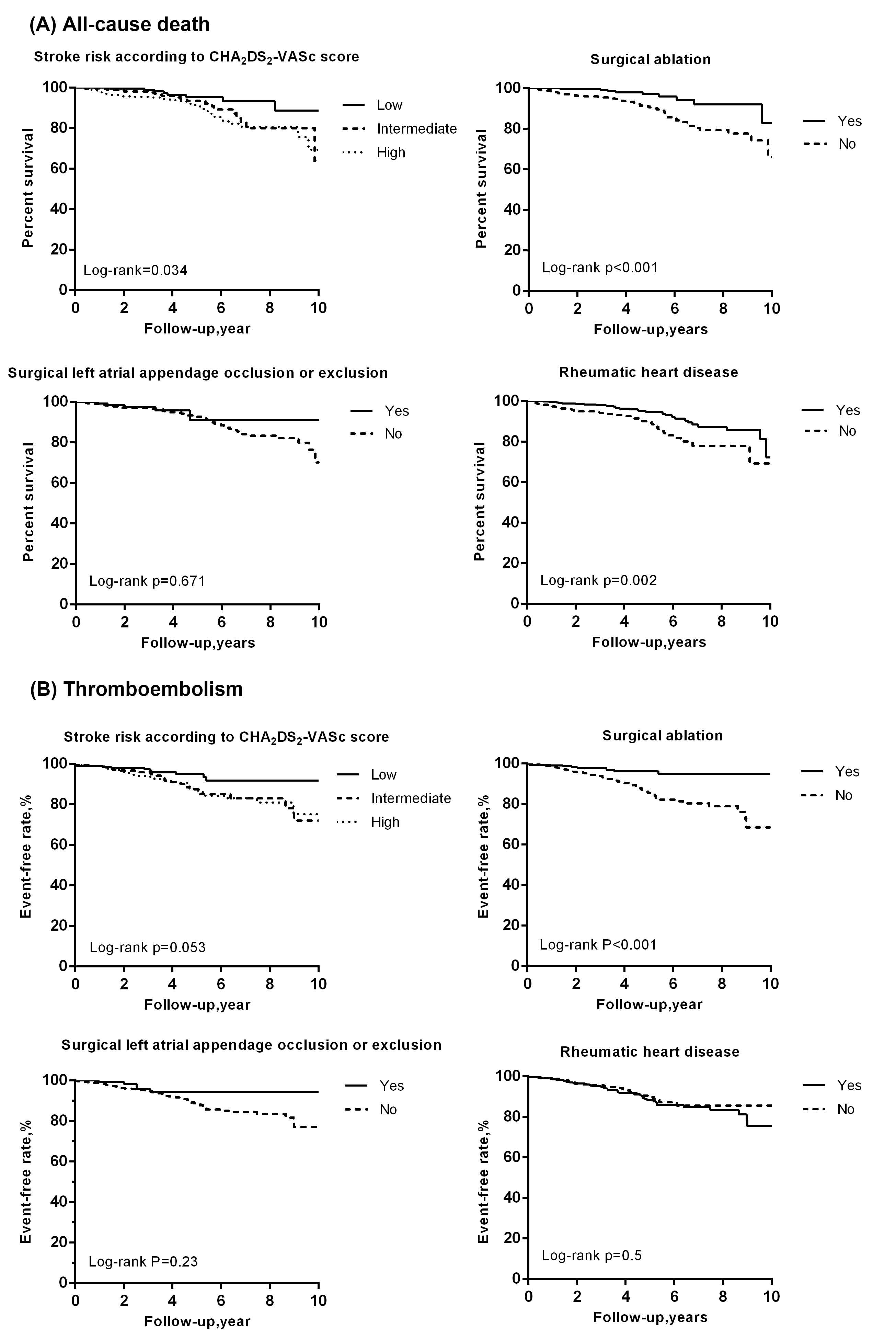

Supplement: Supplementary Figure 1 — The event-free survival of the patients in the different groups (n = 903). (A) All-cause death; (B) Thromboembolism. [file Image_1.JPEG]

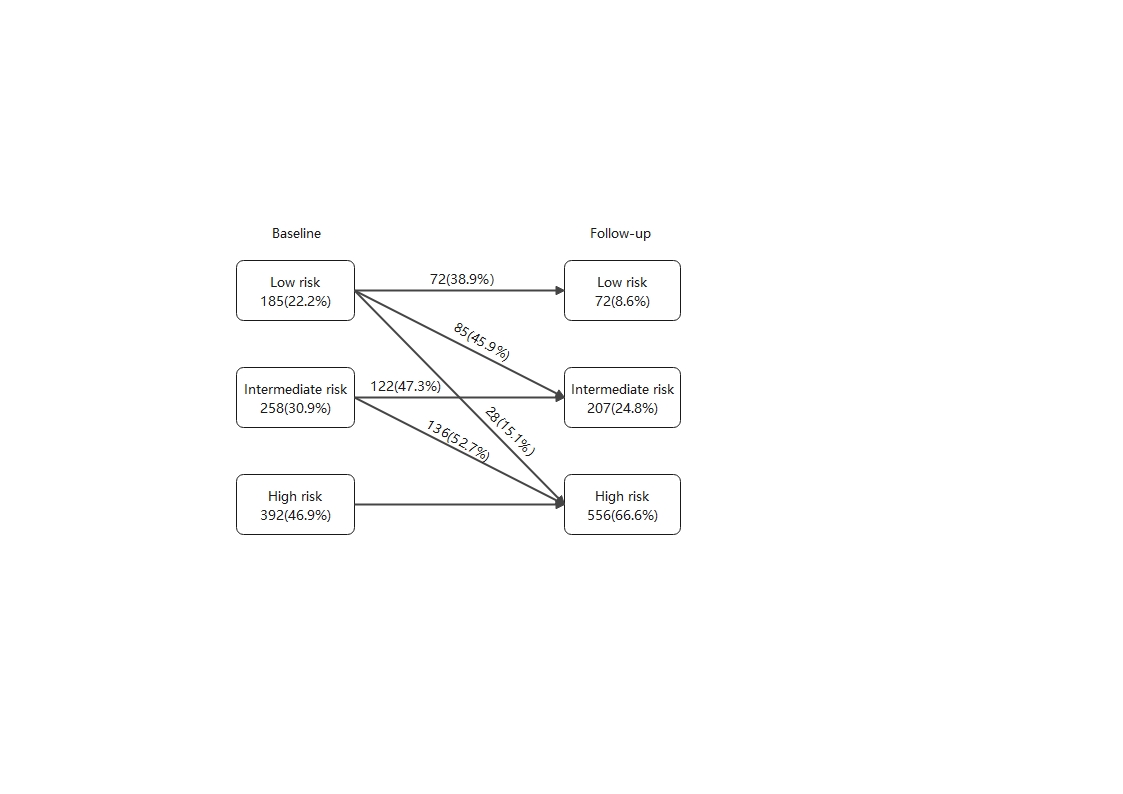

Supplement: Supplementary Figure 2 — The change of the stroke risk category (n = 835). [file Image_2.JPEG]
